# Evaluation of Treatment Effect of Sirolimus on Pediatric Pulmonary Vein Stenosis Using a Neonatal Rat Model

**DOI:** 10.1016/j.jacbts.2025.01.010

**Published:** 2025-04-02

**Authors:** Debao Li, Yingying Xiao, Peisen Ruan, Zunmin Wan, Yuqing Hu, Sijuan Sun, Zheng Wang, Sixie Zheng, Yi Yan, Hao Chen, Hao Zhang, Chun Shen, Qi Sun, Lincai Ye

**Affiliations:** a Department of Thoracic and Cardiovascular Surgery, Shanghai Children’s Medical Center, Shanghai Jiao Tong University School of Medicine, Shanghai, China; b Department of Pediatric Surgery, Children’s Hospital of Fudan University, National Children’s Medical Center, Shanghai, China; c Department of Thoracic and Cardiovascular Surgery, Shanghai Children’s Hospital, Shanghai Jiao Tong University School of Medicine, Shanghai, China; d Department of Critical Care Medicine, Women and Children's Hospital of Ningbo University, Ningbo, Zhejiang, China; e Sichuan Provincial Key Laboratory for Human Disease Gene Study, Genome Sequencing Center, Department of Laboratory Medicine, Sichuan Provincial People's Hospital, School of Medicine, University of Electronic Science and Technology of China, Chengdu, China; f Department of Cardiology, Shanghai Children’s Medical Center, Shanghai Jiao Tong University School of Medicine, Shanghai, China; g Department of Pediatric Intensive Care Unit, Shanghai Children’s Medical Center, Shanghai Jiao Tong University School of Medicine, Shanghai, China; h Shanghai Institute for Pediatric Congenital Heart Disease, Shanghai Children’s Medical Center, Shanghai Jiao Tong University School of Medicine, Shanghai, China; i Institute of Pediatric Translational Medicine, Shanghai Children’s Medical Center, Shanghai Jiao Tong University School of Medicine, Shanghai, China

**Keywords:** congenital heart disease, pediatric, pulmonary hypertension, pulmonary vein stenosis, right ventricle, sirolimus

## Abstract

A modified neonatal rat model of pulmonary vein stenosis (PVS) via bilateral pulmonary vein banding was introduced for the first time, providing a platform for understanding the pathophysiology of PVS and developing treatments. In addition, using this model, the mTOR inhibitor sirolimus was evaluated as a treatment for PVS and exhibited significant therapeutic effects, indicating the need for accelerated ethics approval in clinical settings where sirolimus is not yet in use.

## Highlights

• Bilateral PV banding with a larger but stenosed PV lumen produces similar pathophysiological alterations to those seen in pediatric PVS.
• Sirolimus has a good treatment effect on PVS, indicating a need for accelerated ethics approval in communities where sirolimus is not yet in use.
• This article provides a reader-friendly PVS model construction protocol with schematic diagrams and a surgical video.

## Visual Abstract

**Figure:**
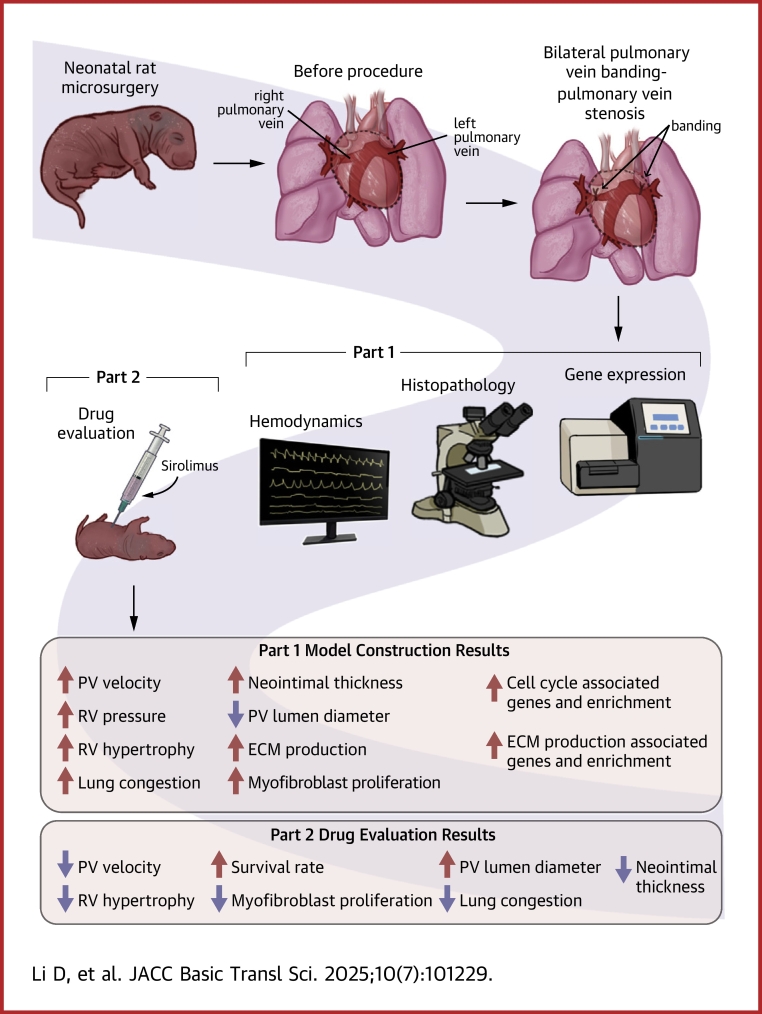

Pulmonary vein (PV) stenosis (PVS) is 1 of the most difficult clinical challenges in the field of pediatric congenital heart diseases that usually leads to secondary pulmonary hypertension and right ventricle (RV) failure.^1^^,^^2^ Within 2 years of diagnosis, the mortality rate of treatment-naive pediatric PVS patients is as high as 60%.^1^^,^^2^ Importantly, the treatment of pediatric PVS is extremely challenging due to the following 2 most important characteristics of PVS: 1) extensive hyperplasia of PV intima, extending from upstream veins to downstream veins; and 2) extremely high recurrence of stenosis after surgical or stent correction, which happens even in the originally normal and unaffected PVs.^1^^,^^2^ These 2 characteristics of PVS have made the search for effective treatments of PVS challenging for over half a century.^1^^,^^2^

Recently, in a clinical trial, the mammalian target of rapamycin (mTOR) inhibitor sirolimus has been shown to increase the survival rate of children with PVS.^1^^,^^2^ However, this clinical trial was limited by its single-center design and small sample size (only 15 patients treated with sirolimus), and the number of repeated stent placements in the sirolimus group was much higher than that in the placebo group, making the clinical promising results of sirolimus controversial.^1^^,^^2^ In addition, the selected patients in the clinical trial had severe PVS. Therefore, it is urgent to test the treatment effects of sirolimus on a small-animal model to provide support for further clinical research and its broader use in PVS treatment.

Diseased animal models play an extremely important role in preclinical research.^3^ Previously, we reported the world's first neonatal rat PVS model.^4^ Specifically, we used unilateral PV banding to construct the model.^4^ However, approximately 30% of the neonates developed PV occlusion, resulting in the formation of collateral circulation (Supplemental Figure S1). In the current study, we modified the model by bilateral PV banding (BPVB) with a larger but stenosed PV lumen, which produced stable PV stenosis, with <10% of rats developing collateral circulation. Using this model, we then evaluated the treatment effect of sirolimus on pediatric PVS.

## Materials and methods

### Rats

We used Sprague-Dawley pups bred in our laboratory for this study. The room was maintained with a 12-h/12-h day/night cycle at a temperature of 21 to 23 °C. All the procedures in this study conformed to the principles outlined in the Declaration of Helsinki and were approved by the Animal Welfare and Human Studies Committee at Shanghai Children’s Medical Center (institutional review board approval no. SCMCIRB-Y2020094).

### Experimental design

The neonatal BPVB surgery comprised the following 3 steps: preoperative preparation, BPVB surgery, and postoperative care. There were 2 experiments. The first was to evaluate the BPVB model, which included 30 rats (15 rats per group). The second was to evaluate the treatment effects of sirolimus, which included 120 rats (60 rats per group).

### Preoperative preparation

**Figure 1:**
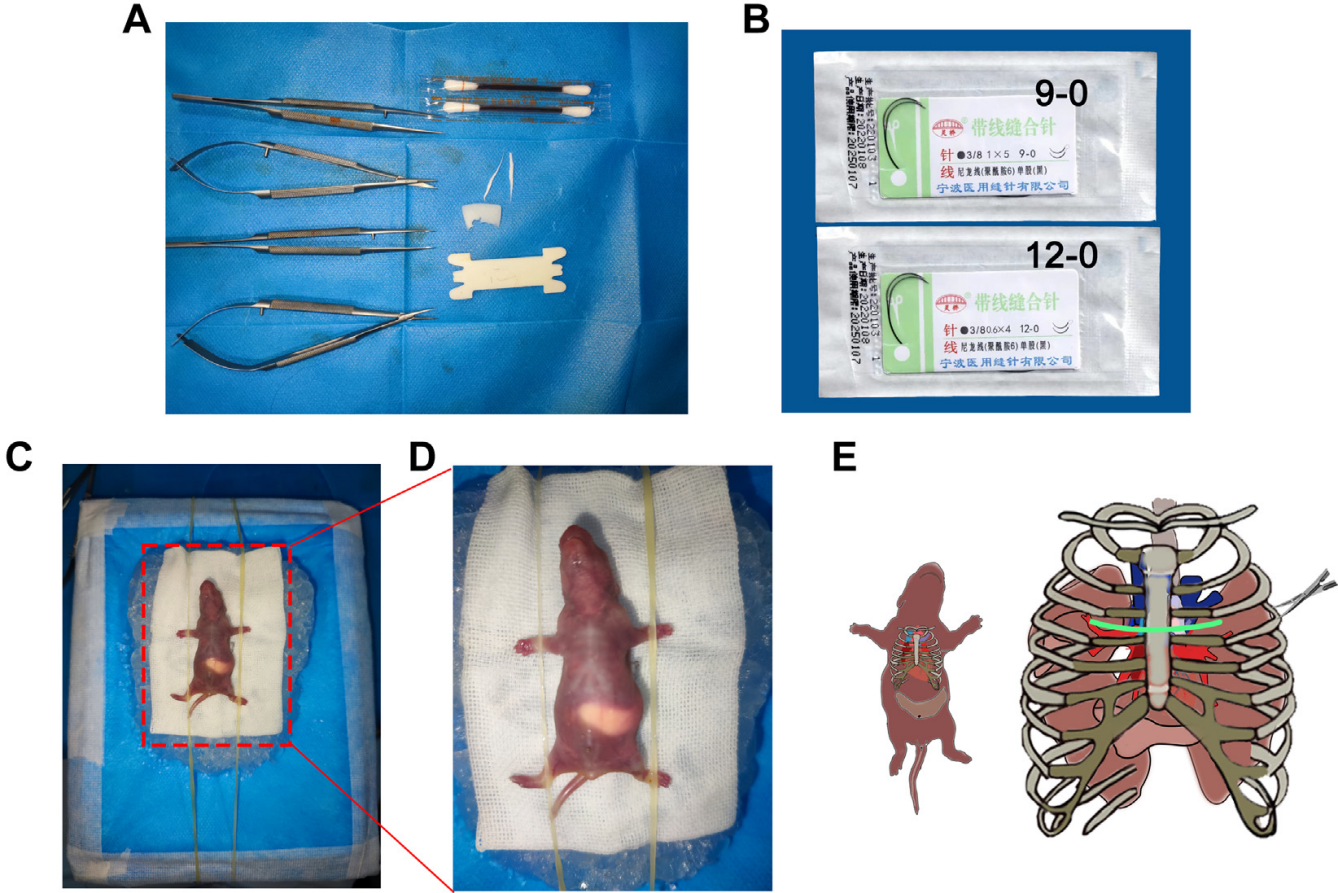
Surgery Setup (A and B) Surgical tools and materials required for neonatal bilateral pulmonary vein banding. (C and D) Neonatal rat immobilized on an ice bed. (E) Schematic exposing pulmonary veins between the left third and fourth ribs (green line) with minimal incision.

The preoperative preparation was similar to our previous study.^3^^,^^4^ Briefly, surgical items were prepared in advance, including 28G padding needles with a 0.18-mm inner diameter (previously, we used 30G padding needles with a 0.16-mm diameter), hemostatic gelatin sponges, cotton balls saturated with iodophor disinfectant, 9-0/12-0 thread, and a water-resistant ice bed (Figures 1A to 1D). The padding needle was cut to a suitable size for placement into the small thoracic cavity. The tip of the 12-0 thread needle was ground to a blunt end to prevent pricking of blood vessels. The ice bed was made from a Styrofoam box enclosed in a sterile towel (Figure 1C and 1D). Two parallel rubber ropes were used to immobilize the rats (Figures 1C and 1D). All surgical instruments were sterilized before surgery to reduce the risk of infection. A schematic demonstrating the exposure of the PVs between the left third and fourth ribs (green line) is presented in Figure 1E to help users become familiar with the anatomical structure of the surgical incisions starting the operation.

### Neonatal BPVB surgery and sham surgery

**Figure 2:**
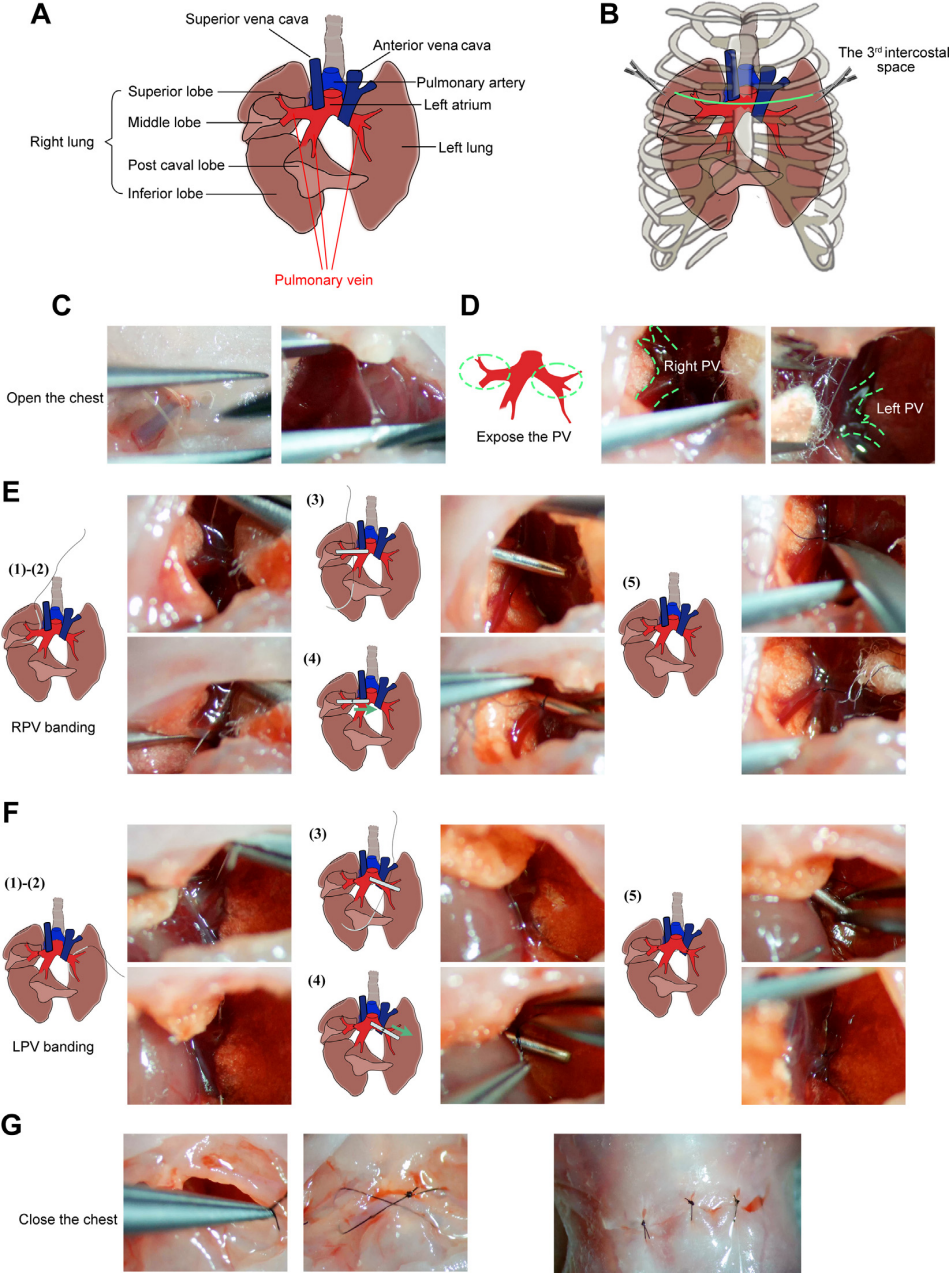
Schematic and Microscopic Views of the Surgical Procedures (A) Schematic diagram of the local anatomical structures around the pulmonary veins (PVs). (B) Schematic diagram of exposed PVs between the left third and fourth ribs (green line) with minimal incision. (C) The sternum was cut, and the chest opened. (D) Exposed left and right PVs. (E) Right PV (RPV) banding: (1) a blunt needle placed beside the RPV; (2) needle passed below the RPV; (3) placement of the 28G padding needle; (4) padding needle ligated with the RPV; and (5) removal of the padding needle and cutting of the end of the sutures. (F) Left PV (LPV) banding: (1) a blunt needle beside the LPV; (2) needle passed below the LPV; (3) placement of the 28G padding needle; (4) padding needle ligated with the LPV; and (5) removal of the padding needle and cutting of the end of the sutures. (G) Layer-by-layer skin closure. Schematic views are shown on the left; microscopic images are shown on the right.

BPVB surgery (Video 1) was performed on the neonatal rats at postnatal days 1 to 3 (P1 to P3). Neonates were anesthetized by direct ice-cooling for approximately 3 to 5 minutes.^3^^,^^4^ The anesthetized neonates were then transferred to an ice bed and fixed in the supine position. A horizontal 5-mm incision was made along the third intercostal spaces (Figures 1E, 2A, and 2B). The pectoralis major and pectoralis minor muscles were separated and cut with microscissors. A retractor was inserted between the incision and used to pull the rib slightly upward to expose a sufficient operating field. Gelatin sponges were used for hemostasis. The pup’s position and retractor were adjusted to clearly expose the PVs used for banding (Figures 2A to 2D, Supplemental Figure S2).

First, we performed right pulmonary vein (RPV) banding surgery (Figure 2E). A 12-0 suture needle was passed through the root of the RPVs connecting the superior and middle lobes (Figure 2E). The needle was held with a needle holder on the opposite side as it was passed through and then gently pulled until the suture thread was completely under the RPV (Figure 2E). The gelatin sponge was removed, the padding needle was placed on the RPV, and the PV and padding needle were ligated with 12-0 thread (Figure 2E). The padding needle was then gently removed using a microholder and a fixed constriction, and a lumen diameter equal to the needle was created (Figure 2E). The end of the sutures was cut (Figure 2E).

Second, we performed left pulmonary vein (LPV) banding (Figure 2F) surgery, which was similar to RPV banding. After RPV and LPV banding, blood was absorbed using cotton swabs, the Gore-Tex patch (Gore Medical) was placed on the gap of the pericardium, the pups were rotated to the transverse position, and the retractor was removed. The chest was then closed layer by layer (Figure 2G). The surgery takes 1 skilled person 15 minutes to complete.

In our evaluation of sirolimus, because the number of neonates was large (120 in total), 4 people operated simultaneously, each responsible for 30 rats. Thus, each person operated for a total of 7.5 h (15 min/rat × 30 neonates = 450 minutes = 7.5 hours). The neonates operated on by the 4 individuals were mixed and then randomly divided into 2 groups at P6 (the BPVB and BPVB + sirolimus groups).

The sham procedure was identical to the BPVB surgery with the exception of the banding step. Specifically, the sham surgery included thoracotomy, pulmonary vein exposure, suture placement and removal, but without banding, and closure of the thoracic cavity.

### Postoperative care

The pups were warmed under a heating lamp set at 40 °C until they could breathe freely (2 to 3 minutes). The pups were then transferred to another heating lamp set at 37 °C until they made natural movements and exhibited red or pink skin (2 to 3 hours).

### Evaluation of sirolimus in the treatment of PVS

Sirolimus (CAS No. 53123-88-9, MCE) or solvent was intraperitoneally injected (1.5 mg/kg/3 days) into the BPVB pups at P7 4 times in accordance with a previous report.^5^ The mortality of each group was recorded blindly from P1 to P30. The rats were sacrificed at P30, and the lungs, hearts, and PVs were harvested for further evaluation.

The effectiveness of the BPVB model was validated by echocardiography, RV pressure, gross anatomical examination, hematoxylin & eosin (H&E) staining, RNA-seq analysis, and immunofluorescence, whereas the effectiveness of sirolimus was evaluated based on gross appearance, RNA-seq analysis, immunohistology, and mortality. All techniques are detailed in the Supplemental Methods. The raw RNA-seq data have been deposited in NCBI’s Gene Expression Omnibus database.^6^

### Statistical analysis

Continuous data are presented in figures using dot plots with the mean ± SD. Differences were tested with Student’s *t*-test if the data were normally distributed; otherwise, they were tested with the Wilcoxon rank sum test. The normality of data distribution was assessed using the Shapiro-Wilk test. Associations between 2 continuous variables were analyzed via Pearson's correlation coefficient analysis, and the log-rank test was used for survival analysis. For RNA-seq analysis, DESeq2 was used to analyze the differentially expressed genes (DEGs), with thresholds set at a q value <0.05 and |log 2(fold change)| >1. Additionally, *P* values <0.05 were considered to indicate statistical significance. Statistical analyses were performed using SAS software version 9.2 (SAS Institute).

## Results

### Neonatal BPVB surgery induces similar pathophysiological alterations to those seen in pediatric PVS

**Figure 3:**
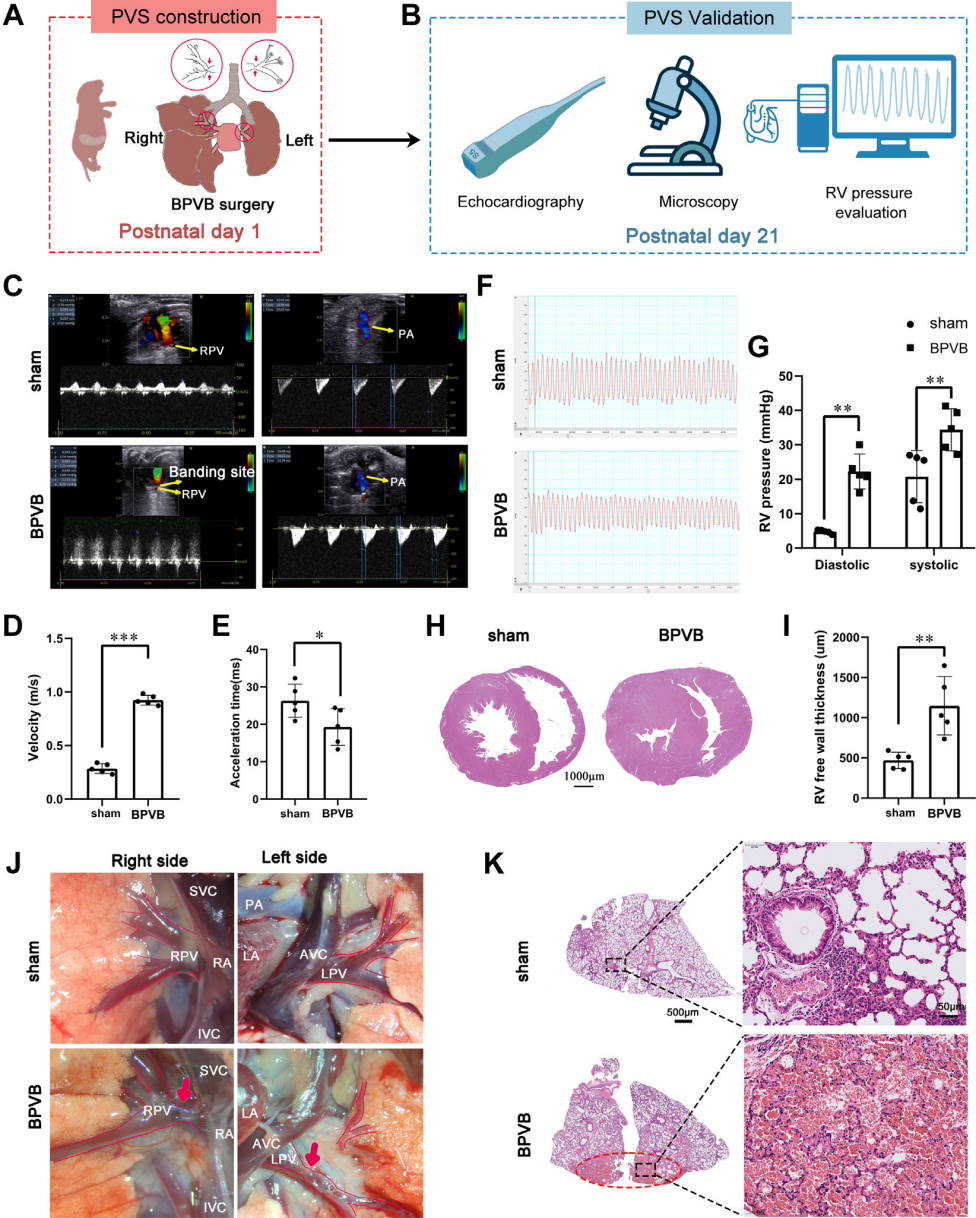
Neonatal BPVB Surgery Induces Pathophysiological Alterations Similar to Those Seen in Pediatric PVS (A and B) Experimental flowchart. (C) Blood flow of PV and pulmonary artery (PA) detected by echocardiography. Yellow arrows indicate the banding site of the right PV; the red dotted line indicates the PA. (D) Quantification of the blood flow velocity across the banding site measured by echocardiography. N = 5 randomly selected rats. (E) Quantification of the PA acceleration time by echocardiography. N = 5 randomly selected rats. (F) Representative plot of right ventricular (RV) pressure in diastolic and systolic periods, and (G) its quantification in the sham and bilateral pulmonary vein banding (BPVB) groups at P21. N = 5 randomly selected rats. (H) Representative hematoxylin & eosin (H&E) staining of the hearts, and (I) the quantification of RV free-wall thickness in the sham and BPVB groups. N = 5 randomly selected rats. (J) Gross appearance of the PVs at postnatal day 21 (P21) after BPVB surgery. The red arrows indicate the stenosis caused by BPVB. (K) Representative H&E staining of the lungs at P21. The red elliptic dotted line indicates the region with pulmonary congestion corresponding to banding PVs. Data are presented as dot plots showing the mean ± SD. Statistical significance was determined using Student’s *t*-test. ∗∗∗*P* < 0.001, ∗∗*P* < 0.01, and ∗*P* < 0.05. AVC = anterior vena cava; LA = left atrium; IVC = inferior vena cava; PVS = pulmonary vein stenosis; RA = right atrium; SVC = superior vena cava; other abbreviations as in Figure 2.

BPVB surgery was typically performed on P1 and validated PVS on P21 (Figures 3A and 3B). Performing BPVB surgery on P3 vs P1 did not result in significant differences in PVS or the death rate. At P21, the BPVB group rats showed significantly higher blood flow velocity at the PV banding sites, lower pulmonary artery acceleration time (indicator of pulmonary arterial hypertension), and higher RV pressure (Figures 3C to 3G) than those of the sham group rats. Consistent with the echocardiography and cardiac catheterization results, RV free-wall thickness significantly increased in the BPVB group (Figures 3H and 3I). The gross anatomical examination and H&E staining assessments showed PV stenosis across the whole PV, including the upstream PV on both sides (Figure 3J). Additionally, pulmonary congestion in the lung lobes corresponded to the banding PVs (Figure 3K). In summary, these results support the successful placement of PV bands with subsequent secondary physiological changes, which include decreased pulmonary artery acceleration time, increased RV pressure and RV free-wall thickness, PV stenosis, and pulmonary congestion, similar to those seen in pediatric PVS.

### Neonatal BPVB surgery induces PV intimal thickening

**Figure 4:**
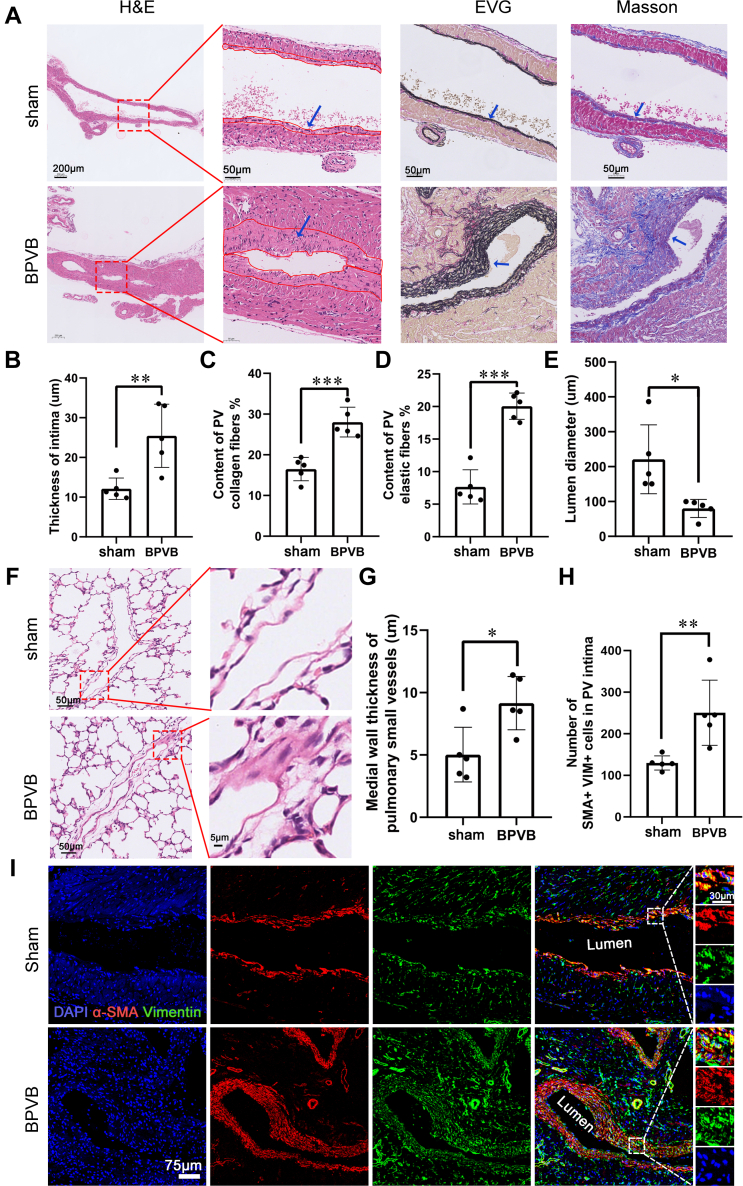
Neonatal BPVB Surgery Induces PV Intimal Thickening (A) Representative H&E, elastin van Gieson (EVG), and Masson staining of upstream PVs in the sham and BPVB groups at P21. The red solid line traces the intima of the PVs. (B) Quantification of the intima thickness. N = 5 randomly selected rats. (C) Quantification of collagen fibers in the intima. N = 5 randomly selected rats. (D) Quantification of elastic fibers in the intima. N = 5 randomly selected rats. (E) Quantification of the PV lumen diameter. N = 5 randomly selected rats. (F) Representative H&E staining of pulmonary small blood vessels in the sham and BPVB groups at P21. (G) Quantification of the medial wall thickness of the pulmonary small blood vessels. N = 5 randomly selected rats. (H) Quantification of the number of myofibroblasts in PV intima per section. N = 5 randomly selected rats. (I) Representative immunostainings of myofibroblasts in the sham and BPVB groups. Cells co-stained with α-smooth muscle actin (α-SMA) (red) and vimentin (green) are myofibroblasts. Most of the α-SMA and vimentin double-positive cells are located in the intima. Data are presented as dot plots showing mean ± SD. Statistical significance was determined using Student’s *t*-test. ∗∗∗*P* < 0.001, ∗∗*P* < 0.01, and ∗*P* < 0.05. Abbreviations as in Figures 2 and 3.

We assessed PV intimal thickening because this is the main characteristic of pediatric PVS.^7^ As shown in Figures 4A to 4D, in the upstream PVs, intimal thickness, collagen fibers, and elastic fibers were all significantly increased in the BPVB group compared with the sham group. Thus, the lumen diameter of PVs was significantly decreased in the BPVB group compared with the sham group (Figure 4E). As shown in Figures 4F and 4G, PV intimal thickening was observed in the lung parenchyma at P21. Immunostaining showed that, in the upstream PVs, the number of myofibroblasts (costained with SMA and vimentin) in intima was significantly higher in the BPVB group than that in the sham group (Figure 4H and 4I). This result suggests that BPVB surgery induces PV intimal thickening, characterized by an increased number of myofibroblasts in the intima.

### Molecular characteristics of thickening PVs

**Figure 5:**
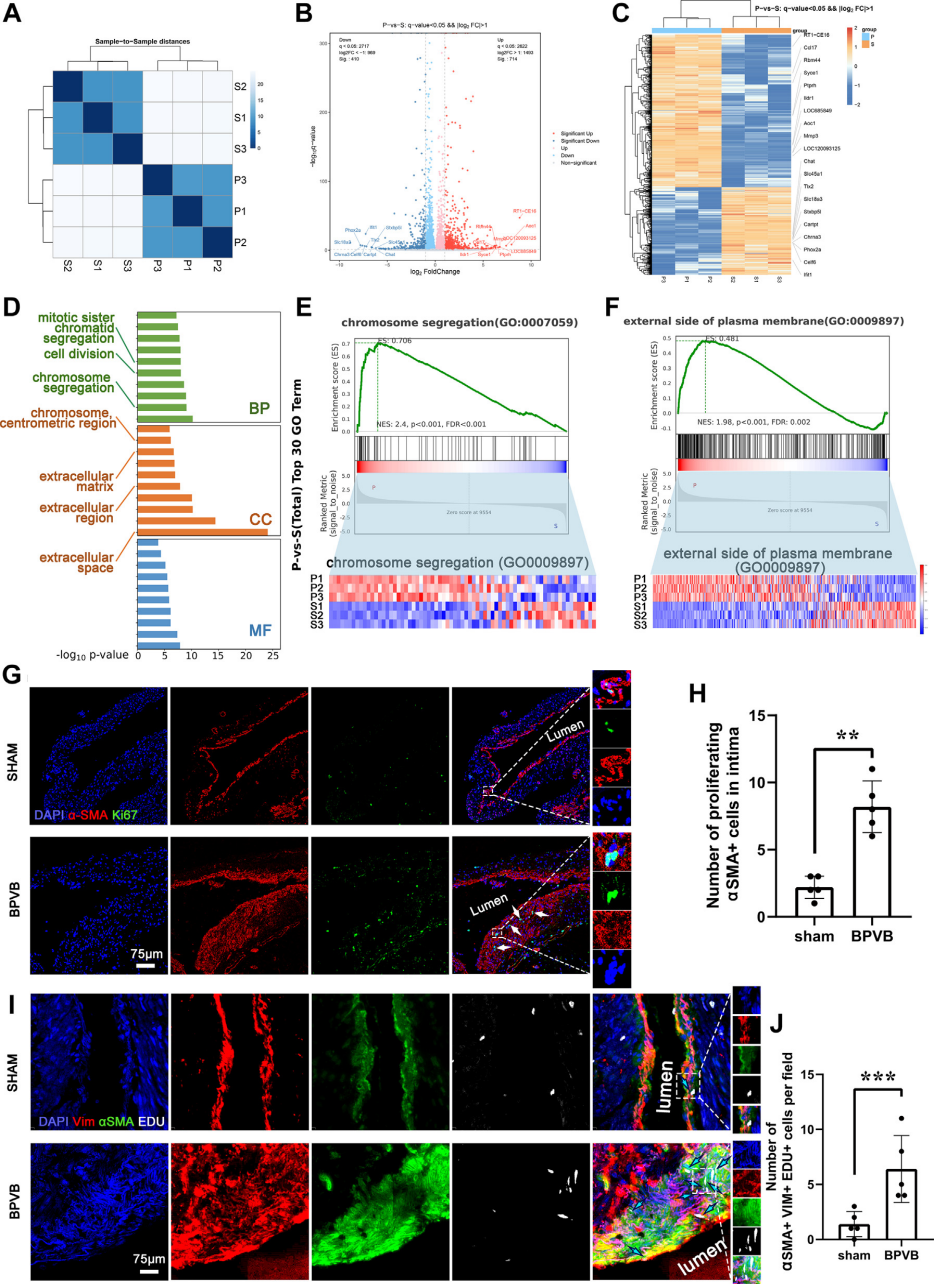
Molecular Characteristics of Thickening PVs (A) Heatmap displaying Euclidean distances between samples based on rlog-transformed data. (B) Volcanic plot of differentially expressed genes (DEGs). (C) Cluster heatmap of DEGs. (D) Top 30 Gene Ontology (GO) terms of DEGs enrichment analysis. (E) Gene set enrichment analysis (GSEA) plot of chromosome segregation and heat map with corresponding DEGs. (F) Gene set enrichment analysis (GSEA) plot of the external side of the plasma membrane and heat map with corresponding DEGs. (G) Representative immunostaining of proliferating α-SMA–positive cells in the sham and BPVB groups. Ki67 (green); α-SMA (red); DAPI (blue). White arrows indicate the proliferating α-SMA–positive cells. (H) Quantification of the number of proliferating α-SMA–positive cells in the pulmonary vein intima per section. N = 5 randomly selected rats. (I) Representative immunostaining of proliferating myofibroblasts in the sham and BPVB groups. α-SMA– (green); VIM (vimentin) (red); DAPI (blue); 5-ethynyl-2’-deoxyuridine (EDU) (White). White arrows indicate the proliferating α-SMA–positive cells. (J) Quantification of the number of proliferating myofibroblasts in pulmonary vein intima per section. N = 5 randomly selected rats. Data are presented as dot plots showing mean ± SD. Statistical significance was determined using Student’s *t*-test. ∗∗∗*P* < 0.001 and ∗∗*P* < 0.01. Abbreviations as in Figures 3 and 4.

Bulk RNA-seq demonstrated that the mRNA expression levels of PV intimal thickening induced by BPVB are stable, showing high intergroup differences and high intragroup consistency (Figure 5A). There were 1,124 DEGs between the 2 groups, 410 with down-regulated expression and 714 with up-regulated expression (Figures 5B and 5C). The enrichment analysis of the DEGs between the BPVB and sham groups demonstrated that the proliferation and extracellular matrix (ECM) gene ontology (GO) terms were among the top 30 enriched terms (Figures 5D to 5F). The bulk RNA-seq results were further confirmed by the immunostaining results, which showed a significantly higher number of proliferating α-SMA–positive cells (Figures 5G and 5H) and myofibroblasts (Figures 5I and 5J) in the PV intima. These results suggest that PV thickening induced by BPVB is characterized by proliferating myofibroblasts, consistent with the findings of a large clinical pathological study.^7^ Figures 4 and 5 support PV intraluminal proliferation and thickening.

### Treatment effect of sirolimus on pediatric PVS

**Figure 6:**
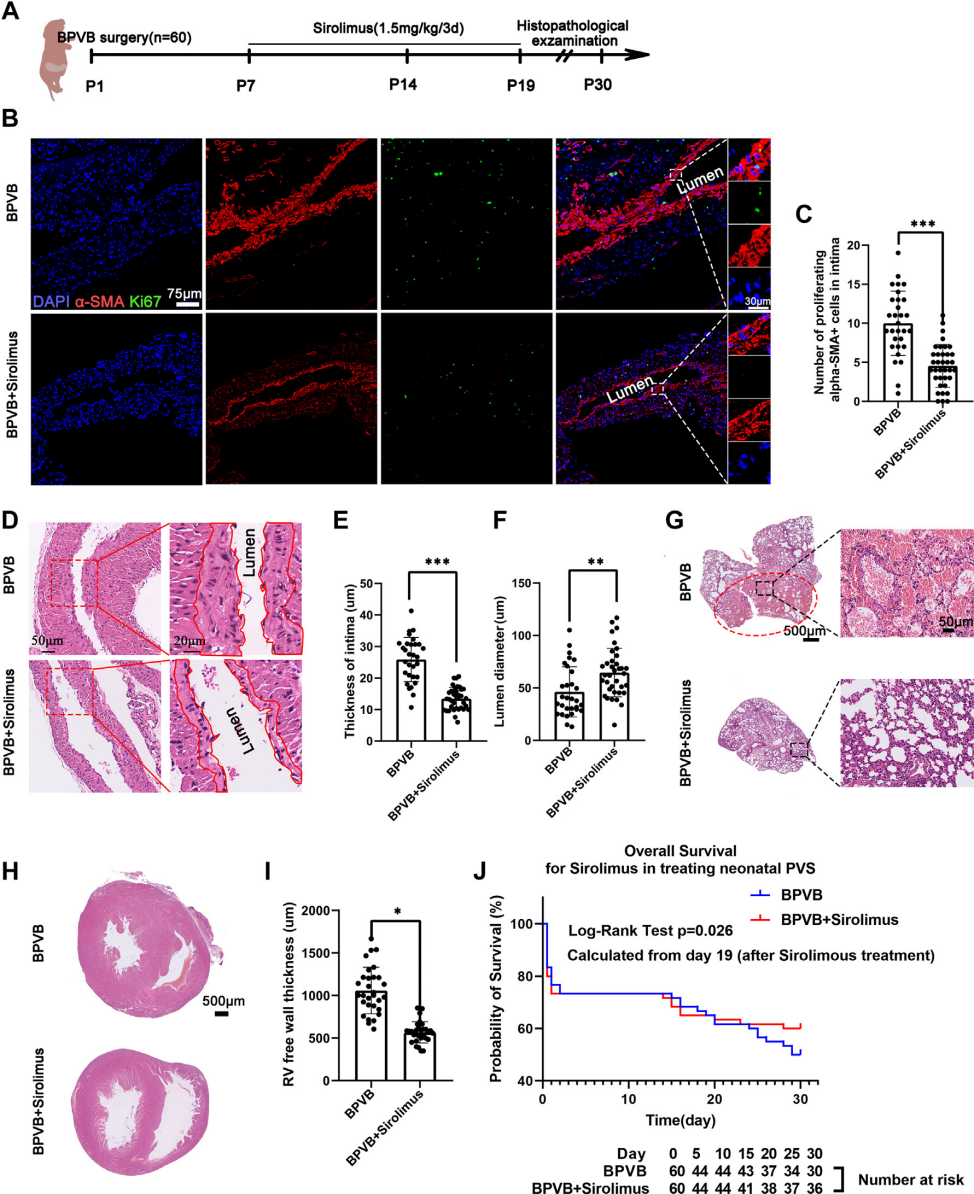
Treatment Effect of Sirolimus on Pediatric PVS (A) Study design and timeline of sirolimus evaluation experiments. (B) Representative immunostaining of the proliferating α-SMA–positive cells in the BPVB and sirolimus-treated groups. Ki67 (green); α-SMA (red); DAPI (blue). (C) Quantification of the proliferating α-SMA–positive cells in the pulmonary vein intima in the BPVB and sirolimus-treated groups. N = 30 rats in the BPVB group; n = 36 rats in the sirolimus-treated group. (D) Representative H&E staining of upstream PVs in the BPVB and sirolimus-treated groups. The red solid line traces the intima of the PVs. Quantification of the intima thickness (E) and lumen diameter (F). N = 30 rats in the BPVB group; n = 36 rats in the sirolimus-treated group. (G) Representative H&E staining of lung lobes in the BPVB and sirolimus-treated groups. The red elliptic dotted line indicates the region with pulmonary congestion corresponding to banding PVs. (H) Representative H&E staining of the heart sections, and (I) the quantification of RV free-wall thickness in the BPVB and sirolimus-treated groups. N = 30 rats in the BPVB group; n = 36 rats in the sirolimus-treated group. (J) Survival curves of the BPVB and sirolimus-treated rats. Data are presented as dot plots showing mean ± SD. Statistical significance was determined using Student’s *t*-test. ∗∗∗*P* < 0.001, ∗∗*P* < 0.01, and ∗*P* < 0.05. Abbreviations as in Figure 2, Figure 3, Figure 4.

There is substantial controversy regarding the use of sirolimus due to the small sample size and severity of cases;^1^^,^^2^ thus, we increased the number of neonatal rats to 60 per group to evaluate the treatment effect of sirolimus on pediatric PVS. Performing 120 neonatal rat BPVB surgeries involved considerable work, taking 4 skilled persons 3 days (Figure 6A). The data showed that, compared with the BPVB group, after sirolimus treatment, the number of α-SMA–positive cells decreased from 10 ± 4.13 to 4.56 ± 2.74 (Figures 6B and 6C) (*P* < 0.001), intimal thickness decreased from 25.9 ± 7 μm to 13.46 ± 3.57 μm (Figures 6D and 6E) (*P* < 0.001), lumen diameter increased from 46.25 ± 23.92 μm to 64.59 ± 23.13 μm (Figures 6D to 6F) (*P* = 0.002), lung congestion decreased (Figure 6G), and RV free-wall thickness decreased from 1,058.78 ± 273.21 μm to 544 ± 135.42 μm (Figures 6H and 6I) (*P* = 0.010). Consequently, the survival rate of the neonatal BPVB rats was increased (Figure 6J). We noticed that there was a considerable overlap of the number of α-SMA–positive cells/intima thickness/lumen diameter between the 2 groups. There are 3 possible reasons for this phenomenon: the variability of biological samples, individual differences in drug sensitivity, and errors in experimental procedures.

## Discussion

Due to the challenging nature of PVS treatment and extremely high mortality rate, physicians are generally pessimistic about the prognosis of children with PVS;^8^^,^^9^ thus, treatment methods for PVS are referred to as a “Holy Grail.”^1^^,^^2^ To search for treatment for PVS, Boston Children's Hospital (1 of the world's top children's hospitals) spent nearly 20 years collecting PVs from 97 children with PVS for pathological examination and identified PVS-unique myofibroblasts that they believe to be the culprit of PVS.^7^ However, what are the unique markers of these myofibroblasts that distinguish them from others? Where do they originate from? Furthermore, are the myofibroblasts an initiating cause of PVS or the downstream effector cells? There are no answers to these questions or paths to find the answers. However, it is known that the myofibroblasts are characterized by high proliferation; thus, for patients with severe PVS, physicians have adopted chemotherapy drugs, such as methotrexate and vinblastine, to inhibit myofibroblast proliferation. Unfortunately, methotrexate and vinblastine are ineffective and cause serious toxic chemotherapy-associated side effects.^10^^,^^11^ Although imatinib is the first-line treatment for PVS prevention at 2 large referral centers,^12^ its therapeutic effect has not been widely recognized, and its mechanisms of action also need further clarification.

Animal models of disease are critical for drug evaluation and mechanism exploration.^3^ The first neonatal piglet PVS model induced by PV banding was introduced in 1990.^13^ In 2020, a modified neonatal piglet PVS model induced by cutting and resuturing PVs was further developed.^14^ However, it takes these 2 piglet PVS models at least 8 weeks to develop PVS.^13^^,^^14^ Evaluations of the neonatal piglet models revealed activation of the mTOR pathway, and the mTOR inhibitor rapamycin (sirolimus) delayed PV stenosis in 5 piglets.^14^ Subsequently, in 2021, sirolimus was clinically tested in 15 children with severe PVS, increasing their survival rate.^1^^,^^2^ In addition, sirolimus is used at some centers routinely to reduce in-stent restenosis or as a primary treatment;^5^ however, the use of sirolimus in this context remains controversial.^1^^,^^2^ Evaluation of the treatment effect of sirolimus in small animals would facilitate ethics approval for using sirolimus to treat mild or moderate PVS in a broader clinical community. Using the neonatal rat model of PVS, we demonstrated that sirolimus (1.5 mg/kg/3 days^15^) has a good treatment effect on PVS by inhibiting the proliferation of myofibroblasts, supporting that sirolimus deserves further clinical investigation regarding its use for treating mild/moderate PVS.

Neonatal mouse/rat size and the surgical time complicate neonatal mouse/rat PV banding, which requires skilled microsurgical techniques. Fortunately, we have developed our own microsurgical techniques and used these to construct neonatal mouse/rat surgical models for nearly 10 years.^3^, ^4^, ^16^^,^^17^, ^18^, ^19^, ^20^ In 2023, we reported the world’s first neonatal rat model of PVS induced by unilateral PV banding.^3^ Here, we introduced a modified and more stable neonatal rat model of PVS induced by BPVB. We have additional tips for successful PVS model construction: 1) use laboratory-bred pups rather than purchased pups as cannibalization can occur when the mother becomes frightened during transportation; 2) use an experienced mother rather than a primipara; and 3) return all the pups together as a group to their mother.

The time to master neonatal BPVB technology closely depends on whether the learner is a beginner in microsurgery. For individuals who are new to microsurgery, it takes approximately 1 to 2 months to become familiar with the neonatal surgical techniques and the microscopic instruments that are used for operation. By contrast, for those who are skilled pediatric cardiac surgeons or are familiar with microsurgery, it only takes 1 to 2 weeks to become familiar with local anatomical structure for BPVB and to master the skill. In addition, it is recommended that the instructor should use a dual-head microscope to teach the learner because that enables the instructor to guide the learner who is seated beside the instructor on the key technical points while performing the surgery. Once the learner masters the skill, in order to swiftly and successfully complete the entire BPVB surgery, we recommend practitioners to practice BPVB 2 or 3 more times.

However, the use of sirolimus is associated with various adverse events, including insulin resistance and diabetes, glomerular dysfunction and renal failure, dyslipidemia, mucositis, pneumonitis, lymphedema, angioedema, and osteonecrosis,^21^ indicating that patients undergoing sirolimus require close monitoring. Other more specific targets for treating PVS need to be identified. Using our neonatal rat model of PVS, we have also established the world's first PVS single-cell RNA sequencing database, which demonstrated that the myofibroblasts highly express FAP and function in ECM organization (Supplemental Figure S3). Moreover, the PVS single-cell RNA-sequencing data demonstrated that the myofibroblasts potentially originated from the endothelial–mesenchymal transition (EndMT) of newly emerging endothelial cells 2 and 3, which were not expressed in healthy PV (Supplemental Figure S4). We then planned to clarify what induces the occurrence of EndMT and its underlying mechanisms.

It is well established that pulmonary congestion, surgical procedures, and repeated stent placement can all easily trigger inflammation. In addition, 1 of the strategies to delay the recurrence of PVS is to use sutureless technique to reduce inflammation.^1^ These clinical observations indicate that inflammation may be a trigger for EndMT. If so, what type of inflammatory cells account for the occurrence of EndMT? Our data revealed that only macrophages significantly increased, whereas other immune cells (eg, neutrophils, T cells, and B cells) decreased in the PVS group compared with the sham group (Supplemental Figures S5A and S5F). Moreover, ligand–receptor interaction analyses demonstrated that the Angpt1–Tek interaction between macrophages and endothelial cells in the PVS group noticeably increased (Supplemental Figures S5G and S5H). Previous studies have established that Tek activates the downstream PI3K–AKT pathway,^22^^,^^23^ which then up-regulates the zinc-finger transcription factor SLUG.^24^ SLUG up-regulates matrix metalloproteinase MM2/9, causing endothelial cells to sprout and migrate to the interstitium, resulting in EndMT.^25^ How inflammation triggers EndMT will be a topic of our future investigation. We are now developing a neonatal mouse model of PVS to verify the results of single-cell RNA sequencing. In summary, the introduction of our neonatal rat model of PVS may pave the way for finding a treatment for children with PVS.

Perspectives **COMPETENCY IN MEDICAL KNOWLEDGE:** Neonatal bilateral PV banding surgery with a larger but stenosed PV lumen produces pathophysiological alterations similar to those seen in pediatric PVS. Furthermore, sirolimus has shown a good effect in treating PVS, indicating a need for accelerated ethics approval in communities where sirolimus is not yet in use.

**TRANSLATIONAL OUTLOOK:** The introduction of our improved neonatal rat model of PVS paves the way for identifying potential treatments for PVS. Future research should focus on elucidating the causes and mechanisms of EndMT.

## Funding Support and Author Disclosures

This work was supported by the Shanghai Natural Science Foundation (No. 22ZR147900 and 23ZR1441100), the National Natural Science Foundation of China (82270314 and 82200309), the Ningbo Top Medical and Health Research Program (No. 2022020405), the Science and Technology Development Fund of Pudong New Area (PKJ2024-Y13, PKJ2024-Y14, and PKJ2024-Y15), the National Clinical Key Specialty Construction Project (10000015Z155080000004), the Medical and Health Technology Plan Projects of Zhejiang (2024YK1568), and the Ningbo Medical and Health Brand Discipline (PPXK2024-06). The authors have reported that they have no relationships relevant to the contents of this paper to disclose.

## Appendix

Supplemental Material

Video 1 Neonatal BPVB Surgery
