# Supplementary material for: Evaluation of Treatment Effect of Sirolimus on Pediatric Pulmonary Vein Stenosis Using a Neonatal Rat Model
Source: JACC Basic Transl Sci. 2025 Apr 2;10(7):101229. doi: 10.1016/j.jacbts.2025.01.010 (PMC12434204; doi:10.1016/j.jacbts.2025.01.010)
Supplement: Supplemental Material [file mmc1.docx]

**Supplemental Appendix for**

**Evaluation of treatment effect of sirolimus on pediatric pulmonary vein stenosis using a neonatal rat model induced by bilateral pulmonary vein banding**

Debao Li^1,2,#^ MD, Yingying Xiao^3,#^ MD, Peisen Ruan^4,^MD, PhD, Zunmin Wan^9,^ PhD, Yuqing Hu^5^ MD, PhD, Sijuan Sun^6^, MD, PhD, Zheng Wang^1^ MD, Sixie Zheng^1,7^ MD, Yi Yan^7,8^, PhD, Hao Chen^1^ MD, PhD, Hao Zhang^1,7,^ MD, PhD, Chun Shen^2^ MD, PhD, Qi Sun ^1,^* MD, PhD, Lincai Ye^1,7,8,^* MD, PhD

^*^**Address for correspondence:**

Lincai Ye, at Institute of Pediatric Translational Medicine, Shanghai Children’s Medical Center, Shanghai Jiao Tong University School of Medicine, 1678 Dongfang Road, Shanghai 200127, China. Tel: + 86-21-64931212; Fax: + 86 21 64931212; E-mail:ylc717@163.com

Qi Sun, at Department of Thoracic and Cardiovascular Surgery, Shanghai Children’s Medical Center, Shanghai Jiao Tong University School of Medicine, 1678 Dongfang Road, Shanghai 200127, China. Tel: + 86-21-64931212; Fax: + 86 21 64931212; E-mail:empyrealheights@163.com.

**Lists of items in the document**

**Supplemental Methods Page 2-5**

**Supplemental Figs Page 3-11**

**Supplemental References Page 11**

**Supplemental Methods**

For evaluation of the BPVB model, in accordance with the ARRIVE guidelines [1], and to reduce the use of animals, we randomly selected five cases per group for echocardiography, catheterization, H&E staining, and immunofluorescence and three cases for RNA-sequencing analysis. The random selection method was used as follows: after Sham and BPVB surgery, each group had 15 rats, which were labeled as 1 to 15. Then, a random number generator was used four times per group for selecting five numbers each time to perform echocardiography, catheterization, H&E staining, and immunofluorescence. Then, we removed the numbers used for H&E and immunofluorescence and randomly selected three numbers from the remaining numbers for RNA sequencing. For the evaluation of sirolimus, the surviving rats were sacrificed, and their lungs and hearts were harvested; then, the heart and lung tissue sections were subjected to H&E and immunofluorescence staining.

**Confirmation of PV obstruction by echocardiography and cardiac catheterization**

At P21/P30, the rats were anesthetized with isoflurane (isoflurane/oxygen: 5% induction, 1.5–2.0% maintenance) and placed on a warming plate in a supine position. The echocardiography measurements were performed via a Vevo 3100 echocardiography system with a 25-MHz transducer in a long-axis view of the main pulmonary artery (PA). Pulsed-wave Doppler was used to measure the pulmonary artery acceleration time (PAAT). The left and right pulmonary vein (PV) velocities were measured and recorded in the apical four-chamber view. The echocardiography was performed by a single experienced physician blinded to the experimental conditions.

Cardiac catheterization was performed to assess the RV pressure on P21/P30. Briefly, the rats were anesthetized via an intraperitoneal injection of 3% pentobarbital sodium (0.1 mL/100 g). After the adequacy of anesthesia was confirmed by testing the toe pinch reflex, the rats were fixed on a foam board, and the jugular vein was carefully dissected. A 4.5-mm conductance catheter (Millar Instruments, Houston, TX) was inserted into the jugular vein and tied with a thread. The catheter was carefully pushed forward until it reached the RV, and the RV pressure waveform was recorded after stabilization. The equipment was calibrated to zero before every measurement was taken. The data were averaged from three continuous waveforms and analyzed blindly.

**RNA-seq**

PV tissues were immersed in TRIzol reagent (Invitrogen, CA, USA) to extract microscale total RNA according to the manufacturer’s instructions. Transcriptome sequencing and analysis were conducted by OE Biotech Co., Ltd. (Shanghai, China). RNA quantification and purity were measured via a NanoDrop 2000 spectrophotometer (Thermo Scientific, USA). RNA integrity was evaluated with an Agilent 2100 Bioanalyzer (Agilent Technologies, Santa Clara, CA, USA). A VAHTS Universal V6 RNA-seq Library Prep Kit was used to construct the libraries according to the manufacturer’s protocol. The libraries were then sequenced on an Illumina NovaSeq 6000 platform, and 150 bp paired-end reads were generated. Fastq format raw reads were first processed via fastp [2], and the low-quality reads were deleted to obtain the clean reads. Approximately 20.9 G of data were generated. The clean reads were mapped to the reference genome via HISAT [3]. The FPKM [4] value of each gene was determined, and the read counts of the genes were calculated via HTSeq-count. PCA was performed via R (v 3.2.0) to measure the biological duplication of each sample. On the basis of the hypergeometric distribution, R (v 3.2.0) was used to perform GO enrichment analysis of the DEGs and construct a column diagram. Gene set enrichment analysis (GSEA) was performed via GSEA software [5]. KEGG datasets were searched for two predefined gene sets, and the genes were ranked according to the degree of differential expression in the two groups. Next, we tested whether the predefined gene sets were enriched at the top or bottom of the ranking lists.

**Single-cell RNA-seq and Analysis**

Single-cell RNA-seq was performed at P21 to analyze the gene expression profiles of individual cells in the pulmonary veins. Pulmonary veins from 20 rats, with 10 in the sham group and 10 in the BPVB group, were harvested for single-cell RNA sequencing. Data were preprocessed using the 10x Genomics Cell Ranger pipeline and aligned to the rat reference genome, Rattus norvegicus, to generate gene count matrices. Each sample underwent independent quality control and doublet removal, applying stringent criteria: nFeature_RNA > 200 and < 7500, nCount > 1000 and < 10000, with mitochondrial gene content below 5%. Doublet identification was carried out using the DoubletFinder (v3.0) algorithm[6]. Following this, the datasets were merged, and batch effects were assessed and corrected using the Harmony algorithm[7], resulting in a total of 23,304 cells with an average gene expression of 1,399 genes per cell.

Cell annotation was performed manually within the Seurat (v4.0) framework[8], utilizing the "FindAllMarkers" algorithm to identify genes specifically expressed within each cell population. Initial annotation involved identifying broad cell subpopulations based on the expression of key marker genes. Epithelial cells, likely originating from the lung parenchyma rather than the pulmonary vein, were excluded from the analysis. After delineating the major cell types, further subclass annotations were conducted for key cell types of interest, such as endothelial cells, immune cells, and myofibroblasts. The analysis also included quantifying changes in cell proportions between experimental groups to investigate their roles in disease progression.

To explore the functional roles of distinct cell populations in disease development, we performed subgroup-specific pathway enrichment analysis by extracting upregulated genes using Seurat’s "FindMarkers" function. This was followed by Gene Ontology-based enrichment analysis using clusterProfiler (v3.16.1)[9]. Additionally, we employed CellChat (v2.1.0)[10] to investigate intercellular communication, focusing on receptor-ligand pairs expressed in more than 10% of cells within a given population. The parameter "population.size = T" was set to ensure that the effect of the proportion of cells in each cell population was taken into account in the probability calculations, and default settings were used for other parameters.

**Histology**

P21/P30 rats were first anesthetized via an intraperitoneal injection of 3% pentobarbital sodium (0.1 mL/100 g) and then euthanized via spinal dislocation. The PVs were exposed and bluntly separated. The gross morphology of the banding area was captured under a Leica M205 FA stereomicroscope (Leica Microsystems, Wetzlar, Germany). The lungs were perfused with 4% paraformaldehyde through the trachea. The hearts were perfused with PBS through right ventricular outflow. Then, the PVs, lungs, and hearts were harvested. The blood was rinsed with PBS. The harvested PVs, hearts, and lungs were fixed with 4% paraformaldehyde (pH 7.4) overnight. Afterward, the hearts and lungs were dehydrated in an ethanol series, embedded in paraffin, and sliced into 5-μm-thick sections. H&E, Masson, and EVG staining were performed according to the manufacturer’s instructions. Briefly, the paraffin-embedded sections were first deparaffinized and hydrated with the following solutions: xylene for 40 min, 100% alcohol for 10 min, and 75% alcohol for 10 min. The sections were then rinsed with water.

For H&E staining, the sections were immersed in hematoxylin solution for 3 to 5 min and then rinsed in water. Acid alcohol was used to differentiate the sections, and then the sections were rinsed again. Ammonia solution was used to stain the sections. After being washed in slowly running tap water, the sections were dehydrated in 85% alcohol and 95% alcohol for 10 min and stained with eosin for 5 min. Finally, they were dehydrated in 100% alcohol for 15 min, cleared in xylene for 10 min, and mounted with resin.

For Masson’s trichrome staining, the tissue sections were immersed for 10 min, repeated twice, and the excess liquid was gently shaken off. The tissue sections were immersed in progressively more dilute ethanol solutions and ultimately immersed in distilled water to rehydrate the tissue: absolute ethanol for 5 min, 95% ethanol for 5 min, 85% ethanol for 5 min, and 75% ethanol for 5 min. Then, the sections were rinsed with distilled water for 1 min, immersed in Bouin’s solution or Zenker’s solution overnight, then rinsed with running water. The sections were subsequently stained with hematoxylin solution (Harris) or iron-containing hematoxylin for 5–10 min and slightly washed with running water. The sections were then differentiated with 0.8–1% hydrochloric acid alcohol and washed with running water for several min. Then, the sections were stained with ponceau acid fuchsin solution for 5–10 min, washed with running water, treated with phosphomolybdic acid solution for approximately 5 min, and stained with aniline blue solution for 5 min without washing. Finally, the sections were treated with 1% glacial acetic acid for 1 min, dehydrated with 95% alcohol several times, dehydrated with absolute alcohol, cleared with xylene, and then mounted with neutral balsam.

For EVG staining, the sections were oxidized with potassium permanganate for 5 min, bleached with oxalic acid for 5 min, and washed with distilled water. Then, the sections were stained with elastin solution for 8–24 h, differentiated in 95% alcohol, and washed with distilled water. The sections were subsequently stained with van Gineon solution for 1 min, rapidly differentiated in 95% alcohol for several seconds, dehydrated with anhydrous alcohol, cleared with xylene, and then mounted with neutral balsam.

For immunofluorescence, the sections were dewaxed in xylene and then rehydrated via an alcohol gradient. The antigen retrieval solution (P0081, Beyotime Biotechnology, Shanghai, PR China) was boiled with the sections for 20 min for antigen repair. The sections were then blocked with blocking solution (PBS with 7.5% goat serum and 0.5% Triton X-100) for 1 h and subsequently incubated with a primary antibody solution overnight at 4°C. The following antibodies were used in the present study: anti-α-SMA (ab7817 Abcam, Cambridge, UK, dilution, 1:200), anti-Vimentin (ab92547, Abcam, Cambridge, UK, dilution, 1:200), and anti-Ki67 (ab15580, Abcam, Cambridge, UK, dilution, 1:200). The sections were warmed at room temperature for 5 min and washed with PBST (PBS with 0.1% Tween 20 (ST1726, Beyotime Biotechnology, Shanghai, PR China)) three times for 5 min each. Then, the sections were incubated with secondary antibodies (Alexa Fluor 488 and/or 555 secondary antibodies (ab150077, ab150078, Abcam, Cambridge, UK; dilution, 1:500)) for 1 h at room temperature. The sections were protected from light after this step. After that, the secondary antibodies were removed, and the samples were washed three times with PBST. The samples were incubated with 4',6-diamidino-2-phenylindole (DAPI, C1005, Beyotime Biotechnology, Shanghai, PR China) for 10 min to stain the nuclei. Neutral balsam and nail polish were used to mount the sections. All the sections were imaged via confocal microscopy. The proliferated intimal myofibroblasts were defined as α-SMA/Vim/EDU- or Ki67-co-positive intima cells to total intima cells (α-SMA positive).

**EDU incorporation**

An EDU Kit (Servicebio, GDP1023) was used to perform the EDU incorporation assay. The EDU powder was diluted with PBS and injected into BPVB or sham rats (5 mg/kg, 1 mg/ml) at P3, P6, and P9. The EDU-traced sections were stained via a click-it EdU-647 kit (Servicebio, G1601) using the same staining methods described above.

**Supplemental Fig.S1**


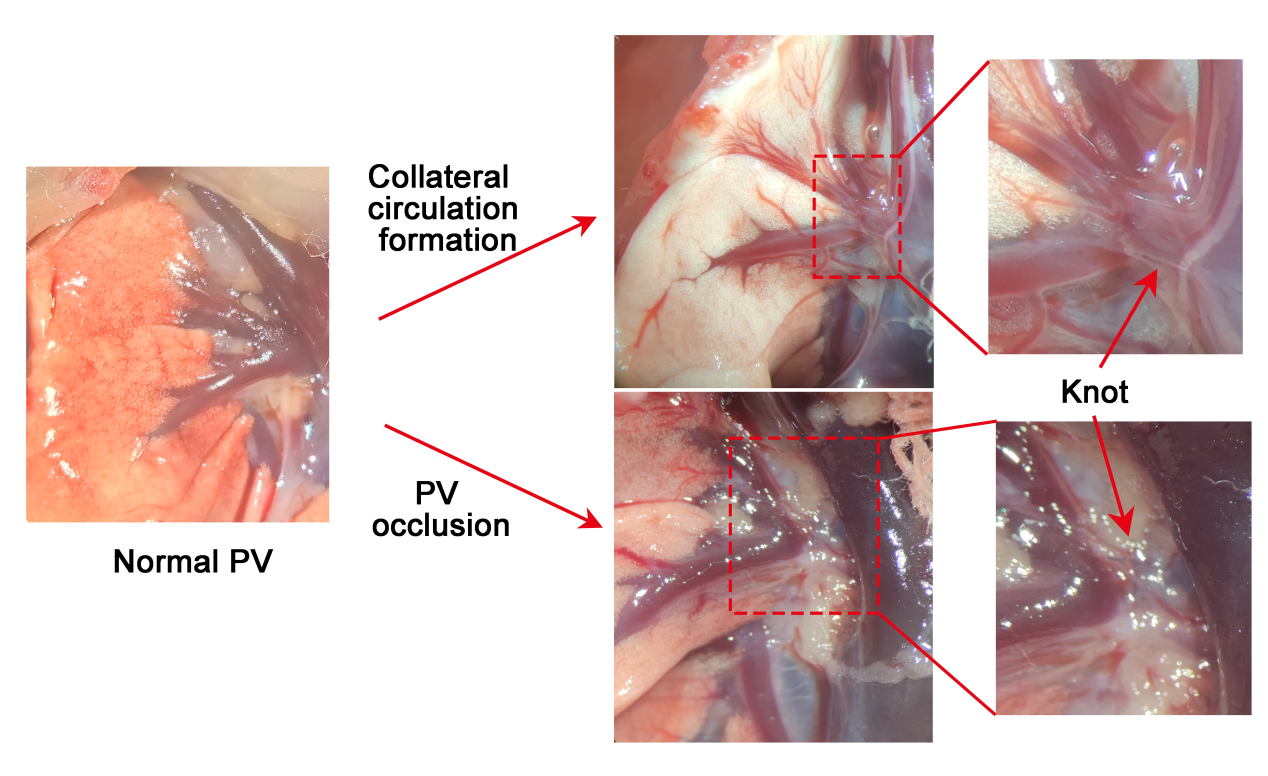


**Supplemental Fig. S1** **PV occlusion and lateral branches formation because of unilateral and more stenosed banding**

**Supplemental Fig.S2**


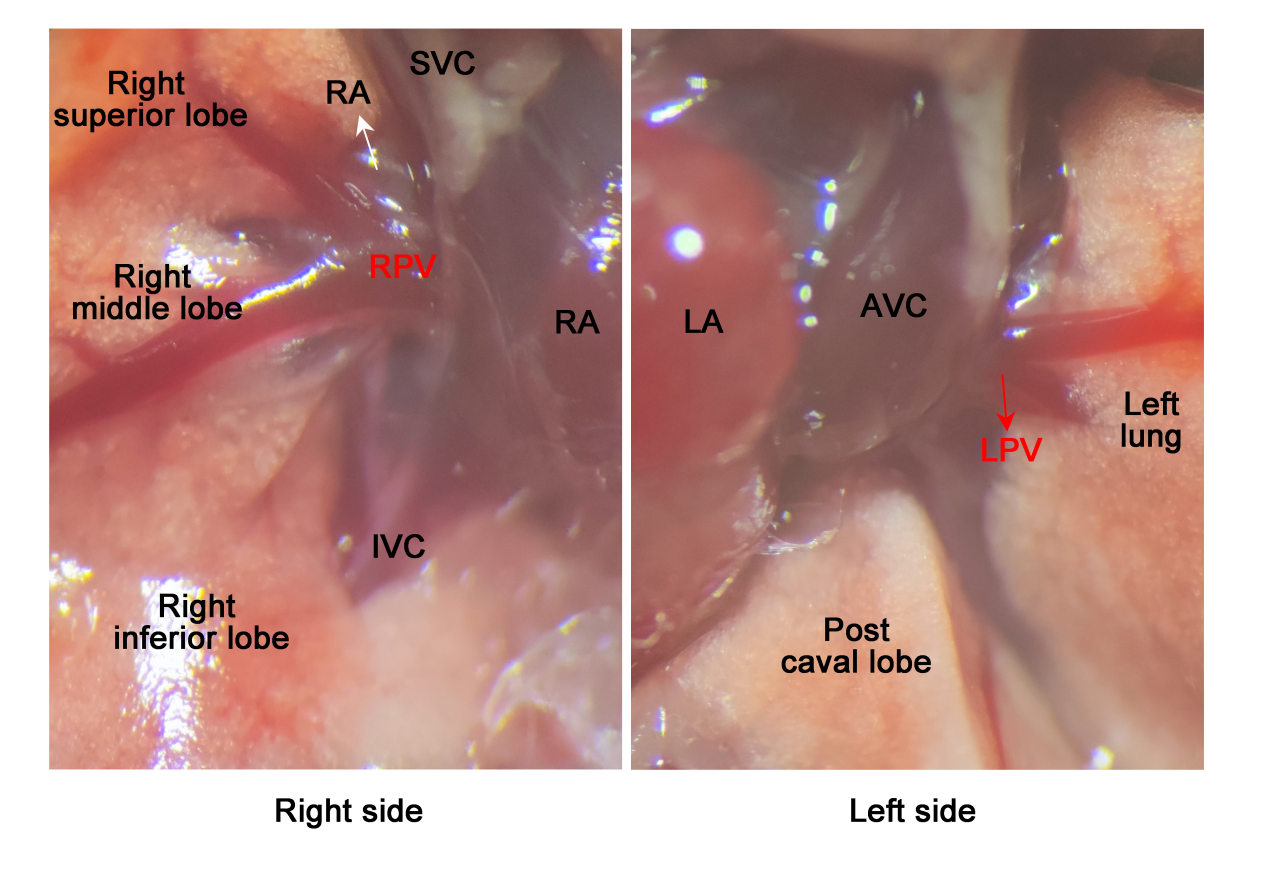


**Supplemental Fig. S2 Right and left PVs used for banding.** SVC: superior vena cava; RPV: right pulmonary vein; RA: right atrium; IVC: inferior vena cava; PA: pulmonary artery; LA: left atrium; AVC: anterior vena cava; LPV: left pulmonary vein.

**Supplemental Fig.S3**


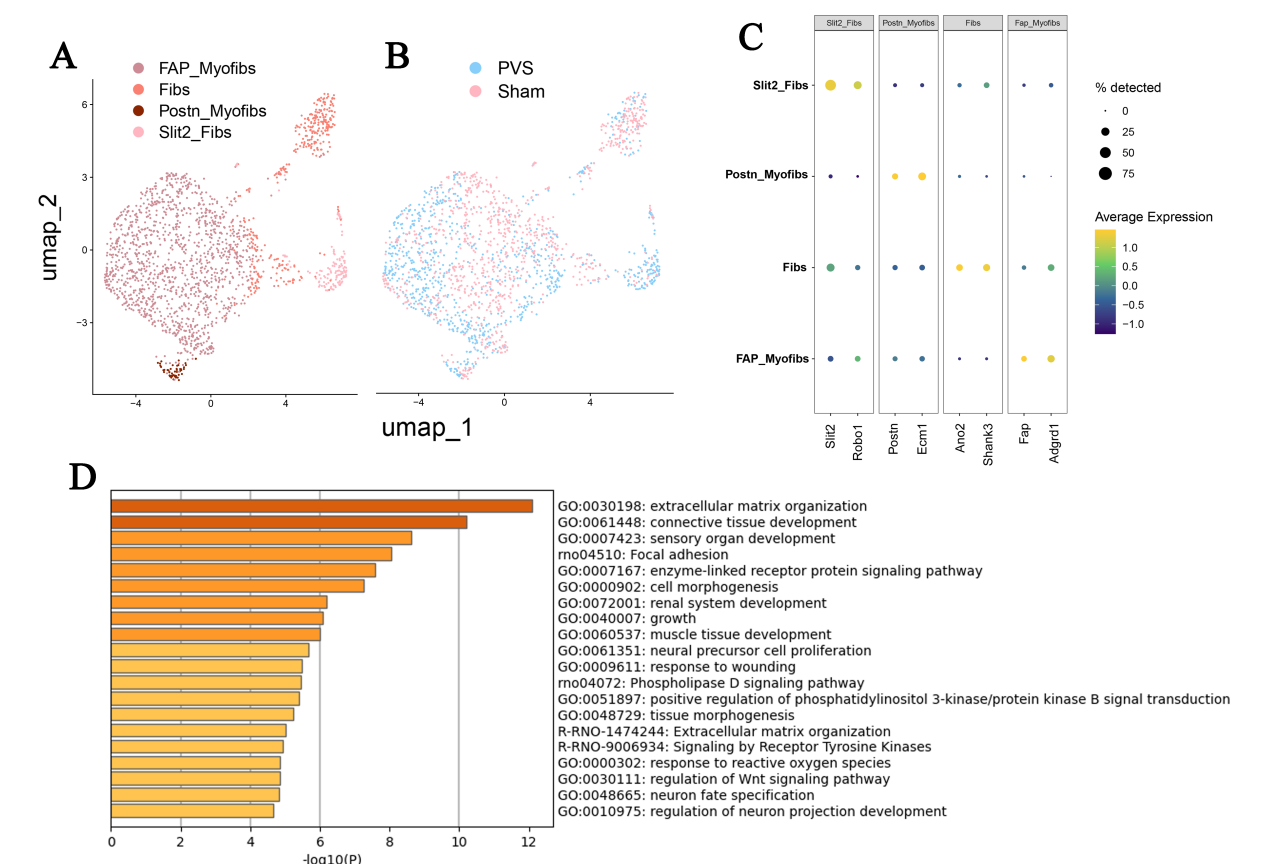


**Supplemental Fig. S3** Markers of the main myofibroblasts that emerge in the PVs of PVS. (A)–(B) Umap analysis of myofibroblasts indicates that FAP_Myofibs are the main myofibroblasts that emerge in the PVs in PVS. (C) Markers of different myofibroblast types. (D) GO enrichment analysis indicating that the major function of FAP_Myofibs is ECM remodeling.

**Supplemental Fig.S4**


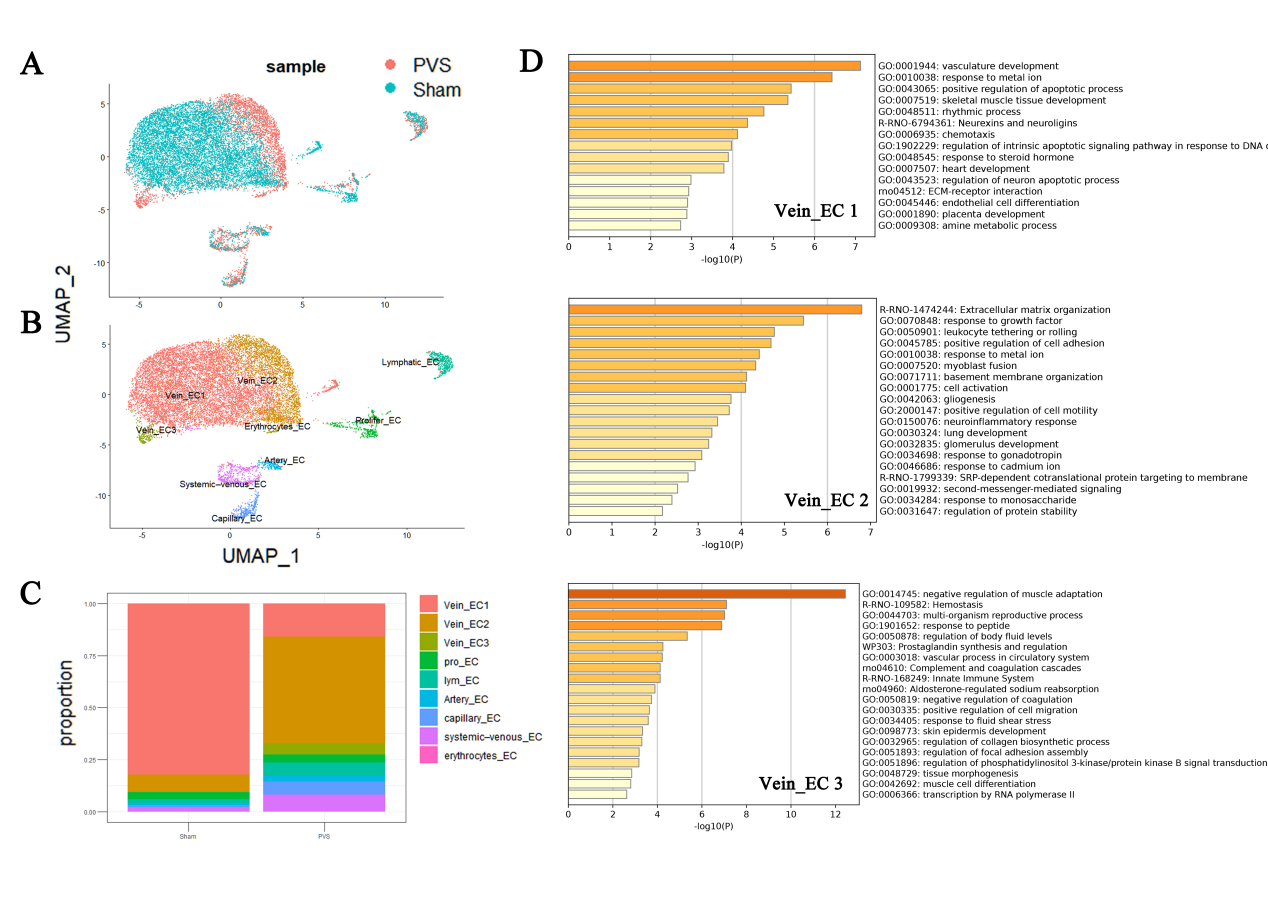


**Supplemental Fig. S4** Newly emerging endothelial cells (ECs) in PVS and their function. (A)–(B) Umap analysis of ECs indicating that EC2/3 are the newly emerging ECs in the PVs in PVS. (D) GO enrichment analysis indicating that EC2/3s function in ECM remodeling and immune responses.

**Supplemental Fig.S5**


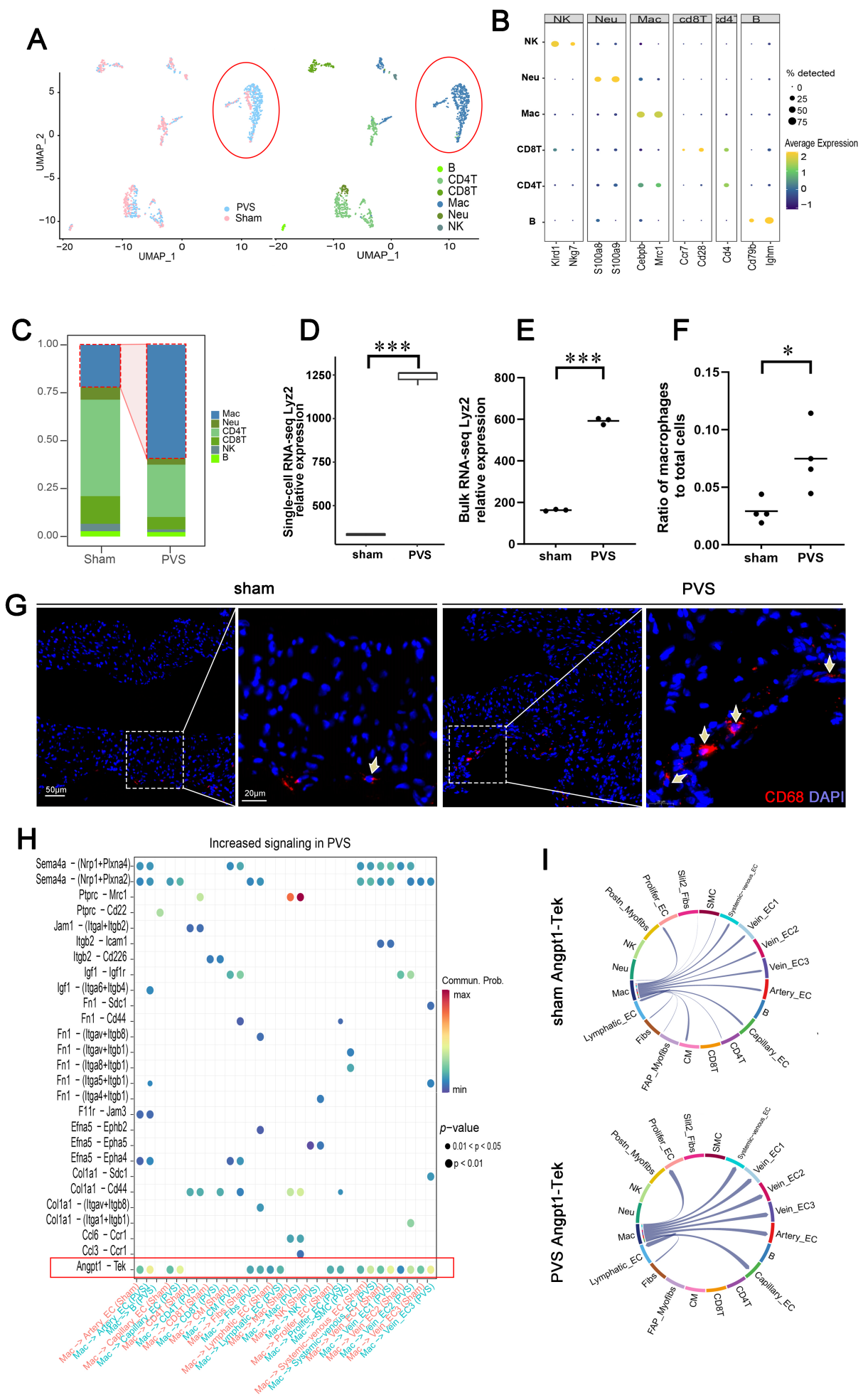


**Supplemental Fig. S5** Angpt1–Tek interaction between macrophages and endothelial cells in PVS. (A)–(B) Umap analysis of ECs indicating that macrophages (Mac) significantly increase in PVS. (C) Single RNA-sequencing analysis demonstrating that the expression of Lyz2 (a marker of Mac) significantly increases in PVS. (D) Bulk RNA sequencing demonstrating that the expression of Lyz2 significantly increases in PVS. (E) Single RNA sequencing demonstrating that the ratio of Mac to total cells significantly increases in PVS. (E) Immunofluorescence staining demonstrating that CD68 (a marker of Mac) significantly increases in PVS. (F)–(G) Single RNA-sequencing analysis indicating that the Angpt1–Tek interactions between macrophages and endothelial cells in PVS are enhanced. Data are presented as dot plots showing the mean ± standard deviation. Statistical significance was determined using Student’s t-test. ***p < 0.001, and *p < 0.05.

**References**

1.Kilkenny C, Browne WJ, Cuthill IC, Emerson M, Altman DG. Improving bioscience research reporting: the ARRIVE guidelines for reporting animal research. PLoS Biol. 2010 Jun 29;8(6):e1000412. doi: 10.1371/journal.pbio.1000412. PMID: 20613859; PMCID: PMC2893951.

2. Shifu C, Yanqing Z, Yaru C, et al. fastp: an ultra-fast all-in-one FASTQ preprocessor[J]. Bioinformatics, 2018, 34(17):i884-i890.

3. Kim D, Langmead B & Salzberg SL. HISAT: a fast spliced aligner with low memory requirements. Nature Methods 2015; 12(4): 357-360.

4. Roberts A, Trapnell C, Donaghey J, et al. Improving RNA-Seq expression estimates by correcting for fragment bias. Genome Biology 2011; 12(3): R22.

5. The Gene Ontology Resource: 20 years and still going strong[J]. Nucleic Acids Res. Jan 2019;47(D1): D330-D338.

6.McGinnis CS, Murrow LM, Gartner ZJ. DoubletFinder: Doublet Detection in Single-Cell RNA Sequencing Data Using Artificial Nearest Neighbors. Cell Syst. 2019 Apr 24;8(4):329-337.e4.

7.Korsunsky I, Millard N, Fan J, et al. Fast, sensitive and accurate integration of single-cell data with Harmony[J]. Nature Methods, 2019, 16(12): 1289-1296.

8.Hao Y, Hao S, Andersen-Nissen E, et al. Integrated analysis of multimodal single-cell data[J]. Cell, 2021, 184(13): 3573-3587.e29.

9.Yu G, Wang L-G, Han Y, et al. clusterProfiler: an R Package for Comparing Biological Themes Among Gene Clusters[J]. OMICS: A Journal of Integrative Biology, 2012, 16(5): 284-287.

10.Jin S, Guerrero-Juarez C F, Zhang L, et al. Inference and analysis of cell-cell communication using CellChat[J]. Nature Communications, 2021, 12(1): 1088.
